# Screening Tools for Early Identification of Adults at High Risk of Type 2 Diabetes: A Scoping Review

**DOI:** 10.3390/healthcare14070839

**Published:** 2026-03-25

**Authors:** Christos Christakis, Dimitra Saliari, Antonis Zampelas, Odysseas Androutsos

**Affiliations:** 1 Laboratory of Clinical Nutrition and Dietetics, Department of Nutrition and Dietetics, University of Thessaly, Argonafton 1C, 42132 Trikala, Greece; cchristakis@uth.gr (C.C.); dimitra.saliari@gmail.com (D.S.); 2 Laboratory of Dietetics and Quality of Life, Department of Food Science and Human Nutrition, School of Food and Nutritional Sciences, Agricultural University of Athens, 75 Iera Odos Street, 11855 Athens, Greece; azampelas@aua.gr

**Keywords:** diabetes mellitus, type 2 diabetes, screening tools, undiagnosed adults

## Abstract

**Highlights:**

**What are the main findings?**

- Fifty-eight screening tools for early identification of adults at high risk for type 2 diabetes mellitus were identified across diverse populations and settings.
- Most tools rely on simple demographic and anthropometric variables, showing moderate to good discriminatory ability with low implementation cost.

**What are the implications of the main findings?**

- Easily applicable screening tools can support large-scale early detection and prevention strategies for type 2 diabetes mellitus, particularly in primary care.
- Integration of validated risk scores into digital and electronic health platforms may enhance population-level screening and reduce healthcare burden.

**Abstract:**

Background/Objectives: Global estimates suggest that approximately 43% of individuals living with diabetes remain undiagnosed, underscoring the need for early identification of adults at high risk of type 2 diabetes mellitus (T2DM) to support timely preventive interventions. This scoping review aimed to map and summarize existing non-invasive screening tools for identifying adults at high risk of T2DM. Methods: PubMed (MEDLINE), Web of Science, ScienceDirect, and Scopus were searched in accordance with the PRISMA extension for Scoping Reviews (PRISMA-ScR). Studies published between 1995 and 2026 that described screening tools for adult populations were included. Results: A total of 58 studies describing screening tools were identified. The tools were developed and applied across diverse populations and ethnic groups. Most were questionnaire-based, easy to administer, and low cost. Commonly included variables comprised demographic characteristics, anthropometric measures, lifestyle factors, and clinical indicators associated with increased T2DM risk. Substantial heterogeneity was observed in tool structure and reported predictive components. Conclusions: This scoping review provides an overview of available screening tools for the early identification of adults at high risk of T2DM. The mapped evidence may inform future validation studies and support context-specific implementation in public health and clinical practice settings, including integration into digital platforms.

## 1. Introduction

Diabetes mellitus (DM) is a serious chronic disease and one of the most important public health challenges worldwide. It is defined as a heterogeneous, multifactorial metabolic disorder characterized by chronic hyperglycemia resulting from defects in insulin secretion and/or action [1]. Type 2 Diabetes mellitus (T2DM), characterized by insulin resistance and progressive β-cell dysfunction [2], accounts for approximately 90–95% of DM cases and primarily affects adults [3]. Because DM may remain undiagnosed for prolonged periods, affected individuals are at increased risk of developing serious long-term complications, mainly due to vascular damage [1]. Early identification of individuals at increased risk is therefore a key component of primary prevention strategies. It is important to distinguish between diagnostic testing, which confirms the presence of diabetes, and screening or risk prediction tools, which aim to estimate the likelihood of developing the disease in asymptomatic populations. Such tools may help reduce the high proportion of undiagnosed cases and support timely lifestyle or clinical interventions.

DM is among the leading causes of mortality worldwide. According to World Health Organization data, diabetes remains among the leading causes of mortality worldwide [4]. The global prevalence of DM has increased steadily over recent decades, rising from 382 million cases in 2013 to 589 million in 2025 [5,6,7]. This upward trend is accompanied by a substantial economic burden on healthcare systems and national economies through both direct and indirect costs [8]. The total global cost of DM has risen dramatically over time, with projections indicating further increases in the coming decades [8]. In addition to system-level costs, individuals and families often experience significant financial hardship related to treatment expenses and productivity losses [9].

Despite growing awareness, a considerable proportion of cases remain undiagnosed. The proportion of diagnosed and treated individuals varies substantially across countries [10]. In high-income settings, up to 30–50% of cases may remain undiagnosed [11], while socioeconomically disadvantaged populations are disproportionately affected [12]. In 2025, approximately 43% of individuals living with DM were unaware of their condition, with particularly high rates of undiagnosed cases reported in low- and middle-income countries [7,13]. Early detection and appropriate management have been associated with improved outcomes in certain populations [14].

Given the rapid development of multiple screening tools over recent decades, along with differences in included variables, validation processes, and application contexts, the existing evidence is characterized by considerable heterogeneity. In such cases, a scoping review is considered an appropriate methodological approach, as it aims to map the extent, range, and characteristics of available evidence rather than to assess comparative effectiveness. Therefore, the purpose of the present study was to identify and summarize the available tools for detecting adults at high risk of developing T2DM.

Specifically, this scoping review sought to address the following research questions:

1. What screening tools have been developed or validated for the early identification of adults at high risk of type 2 diabetes?
2. In which populations and settings have these screening tools been applied?
3. What key characteristics and reported implementation features of these screening tools have been described in the literature?

## 2. Materials and Methods

This study is a scoping review conducted in accordance with the PRISMA Extension for Scoping Reviews (PRISMA-ScR) guidelines. A scoping review approach was chosen to map the available evidence on screening tools for early identification of adults at high risk of type 2 diabetes, given the heterogeneity of study designs, populations, and outcome measures. The PRISMA-ScR checklist is available in the Supplementary Materials (Table S1).

### 2.1. Study Selection Methodology

Studies included in this scoping review were identified through electronic searches of international scientific databases, specifically PubMed (MEDLINE), Web of Science, ScienceDirect and Scopus. The review was conducted in accordance with the Preferred Reporting Items for Systematic Reviews and Meta-Analyses (PRISMA) framework, specifically the PRISMA extension for Scoping Reviews (PRISMA ScR) [15], to ensure transparency and methodological rigor.

### 2.2. Inclusion and Exclusion Criteria

Eligible studies were those reporting on adult populations—either healthy individuals or individuals undiagnosed with T2DM or prediabetes—that described the development or primary validation of screening tools for early risk identification.

The following studies were excluded: (a) studies evaluating or re-validating existing screening tools in populations different from the original derivation cohort; (b) studies involving non-adult populations; (c) studies focusing exclusively on individuals already diagnosed with T2DM; and (d) meta-analyses and systematic reviews. Secondary validation studies conducted in populations other than the original derivation sample were excluded to maintain the focus on identifying and mapping the initial development and core characteristics of screening tools. Inclusion of such studies would have shifted the review toward comparative cross-population performance and effectiveness analysis, which falls beyond the scope of a scoping review.

### 2.3. Search Strategy

The electronic search was performed without time-frame restrictions, covering the period from 1995 to 7 January 2026 to ensure maximum coverage of available data. In addition, a supplementary search was performed in Google Scholar by screening the first 200 results based on title relevance, and the bibliographic references of all eligible studies were screened to identify additional relevant sources, which were reviewed and evaluated using the same methodological approach.

The review questions and eligibility criteria were developed using the Population–Concept–Context (PCC) framework, which is recommended for scoping reviews. This is a well-established methodological approach for formulating research questions and conducting structured searches in the scientific literature (Table 1).

The search strategy was structured according to the Population–Concept–Context (PCC) framework. The Population component included terms referring to adult individuals, such as “adults,” “individuals,” and “undiagnosed”. The Concept component captured terms related to screening and risk assessment tools, including “test,” “risk score,” and “screen*.” The Context component encompassed terms associated with early detection and high-risk identification, such as “prediabetes,” “early diagnosis,” “high risk,” and “predict*.” The database-specific Boolean search strings are provided in Supplementary Table S2.

**Figure 1:**
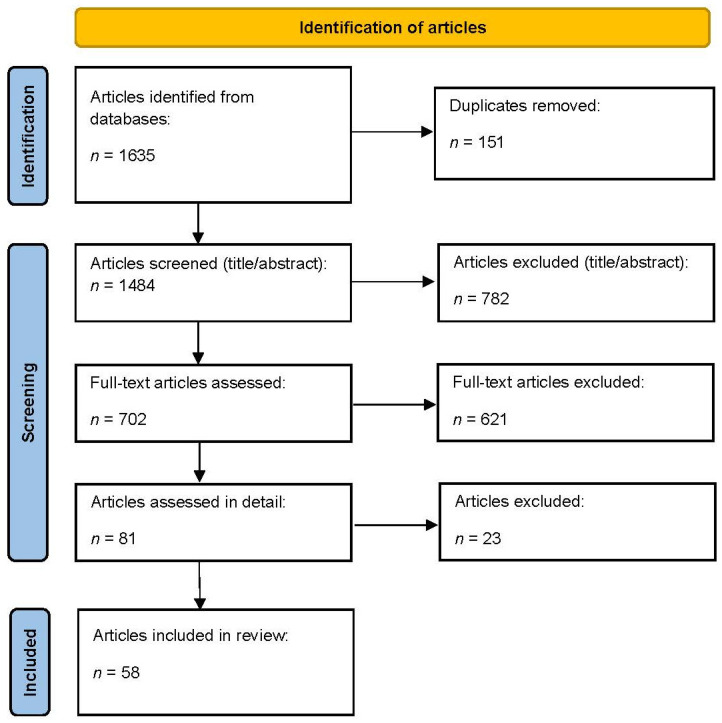
Scoping review flow diagram.

The comprehensive literature search across four electronic databases, namely Scopus (*n* = 987), Web of Science Core Collection (*n* = 278), ScienceDirect (*n* = 149), and PubMed/MEDLINE (*n* = 221), yielded a total of 1635 records for screening. In addition, a supplementary search was performed. No additional eligible records were identified through this supplementary search; therefore, the total number of records included in the PRISMA flow diagram remained unchanged. After screening, 58 articles that met the predefined inclusion criteria were finally included in the review (Figure 1).

### 2.4. Selection and Analysis Process

Study selection was performed independently by two reviewers to enhance transparency and minimize selection bias. Discrepancies were resolved through discussion with a third reviewer. Ιn detail, the studies identified through the search process were assessed for eligibility by two independent researchers (C.C. and D.S.). In the first phase the titles and abstracts were screened to exclude studies that did not meet the inclusion criteria. Studies deemed potentially eligible were then subjected to full-text evaluation. In cases where consensus on study inclusion could not be reached, a third reviewer (O.A.), who was aware of the initial assessments, reviewed the study and made the final decision.

### 2.5. Data Recording

Detailed information from each eligible study was extracted independently by the two researchers using a predefined data extraction table, which was initially piloted on a random sample of studies prior to full data collection. Extracted variables included: (a) key study characteristics (title, authors, year of publication); (b) population characteristics (sample size, geographical location, age range, and proportion of women); (c) components and variables included in each screening tool; (d) validation procedures and reported performance metrics (e.g., AUC, sensitivity, specificity, predictive values); and (e) additional comments or observations.

Data were recorded only when explicitly reported in the original publications, and no attempt was made to impute or estimate missing information. Consequently, variability in reporting completeness across studies is reflected in the descriptive synthesis.

In accordance with established scoping review methodology (PRISMA-ScR framework), a formal risk-of-bias or methodological quality appraisal of the included studies was not conducted. The objective of the present review was to map the breadth, characteristics, and reported features of existing screening tools rather than to critically evaluate internal validity or comparative effectiveness.

## 3. Results

Table 2 presents the 58 screening tools for adults at high risk of developing T2DM, identified based on the predefined study criteria, and recognized according to the screening variables used. For transparency and traceability, Table 2 presents the identified tools in chronological order. However, the instruments may also be conceptually grouped into three broad methodological categories: (i) anthropometric- and questionnaire-based tools, (ii) tools incorporating biochemical parameters, and (iii) algorithmic or machine learning–based models. The outcome targeted by each screening tool (e.g., type 2 diabetes, prediabetes, or impaired glucose regulation) is also presented. Table 3 summarizes the reported accuracy of each tool in the respective studies.

**Figure 2:**
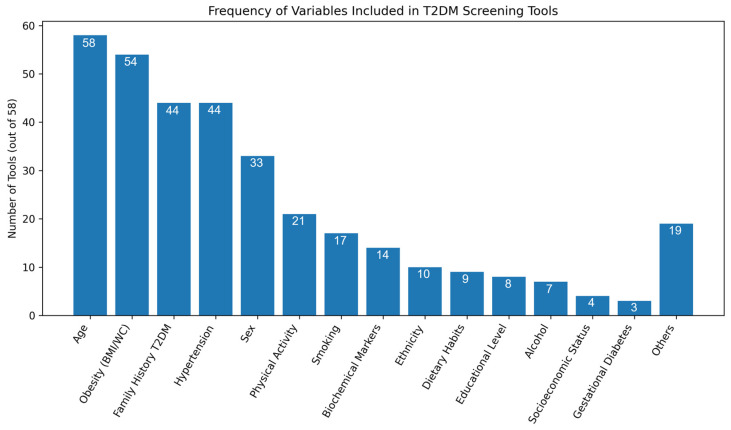
Frequency of variables included across the 58 identified T2DM screening tools, presented in descending order. The “Others” category includes heterogeneous variables reported in fewer studies.

The screening variables utilized across all 58 studies for early identification of T2DM covered a broad range of demographic, anthropometric, lifestyle, and clinical factors. The most frequently used variables were age, sex, body mass index (BMI), obesity, family history of diabetes, hypertension or blood pressure, and waist circumference. Several models also incorporated lifestyle factors such as physical activity, smoking, alcohol consumption, and dietary habits, while others included education, socioeconomic status, and ethnicity. Clinical and biochemical markers, including fasting glucose, lipid levels, glycated hemoglobin (HbA1c), and history of gestational diabetes or hyperglycemia were also considered in some studies (Figure 2).

Performance metrics were reported heterogeneously across studies. Some articles presented discrimination measures such as AUC values, others reported sensitivity and specificity at predefined cut-off points, while a smaller number provided additional indicators including predictive values or calibration measures. The absence of standardized reporting across studies reflects variability in study design and validation objectives rather than selective extraction, and limits direct quantitative comparison between tools. In addition to variability in the type of performance metrics reported, heterogeneity was also observed in threshold selection. In several studies, more than one cut-off value was evaluated, resulting in different sensitivity and specificity profiles. However, detailed justification for the selection of optimal thresholds was not consistently provided.

Across the 58 eligible studies, the accuracy of screening tools for T2DM varied, with area under the curve (AUC) values ranging from 0.68 to 0.92. Using commonly applied thresholds (excellent: AUC > 0.80; good: 0.75–0.80; moderate: 0.70–0.75; limited: <0.70), the majority of tools fell within the good-to-moderate range, with sensitivity and specificity typically between 65–85%. Negative predictive values were consistently high (>90%), whereas positive predictive values remained relatively low. Reported cut-off values also varied across tools and populations.

**Figure 3:**
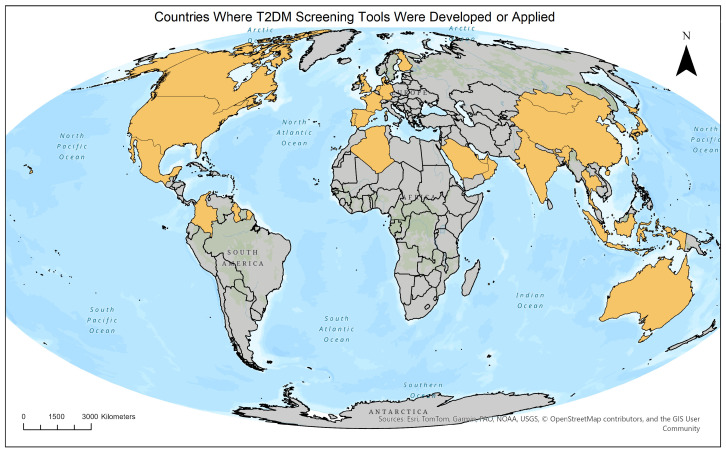
World map showing countries where the identified T2DM screening tools were developed or applied. Highlighted countries correspond to the geographic settings reported in the included studies (Canary Islands are represented under Spain).

The screening tools were applied to populations of diverse nationalities and geographical regions. The highest number of tools was reported in China (n = 12, including Taiwan) and the United States (n = 10). Overall, the tools were tested in 26 countries (Figure 3).

## 4. Discussion

This scoping review mapped the available evidence on screening tools for the early identification of adults at high risk of T2DM. The findings demonstrate substantial heterogeneity in the types of tools identified, the populations and settings in which they were applied, and the characteristics reported across studies. Most tools were simple questionnaires, presented either as decision trees or scoring systems in which the final score indicates the probability of incident T2DM. Many were described as user-friendly and low cost, features that may facilitate their implementation in primary healthcare settings. As reported in the included studies, their primary objective is early risk identification to support preventive strategies.

The identified tools incorporate multiple variables to enhance sensitivity and specificity according to the target population. These variables are generally related to established T2DM risk factors and can be grouped into four broad categories: (a) individual factors (age, sex, BMI, waist circumference, and physical activity); (b) genetic and family factors (family history of DM or hypertension); (c) clinical and metabolic factors (hypertension and gestational diabetes); and (d) socioeconomic and lifestyle factors (educational level, socioeconomic status, dietary habits, alcohol consumption, smoking status, and ethnicity). Age was the only variable included in all screening tools, followed by obesity indicators (BMI and waist circumference) and family history of T2DM, which appeared in 54 and 44 studies, respectively. Hypertension (either as a diagnosis or antihypertensive treatment) and family history of hypertension were also reported in 44 studies, sex in 33 studies, and physical activity in 21 studies. Less frequently included variables comprised ethnicity, biochemical markers, dietary habits, smoking status, and alcohol consumption.

From an implementation perspective, the identified tools differ considerably in feasibility requirements. Questionnaire-based tools relying solely on self-reported demographic and anthropometric variables can be administered rapidly, require no laboratory infrastructure, and are suitable for large-scale community or primary care screening. In contrast, tools incorporating biochemical markers such as fasting glucose or lipid parameters entail additional laboratory costs and logistical considerations, making them more appropriate for structured clinical environments. Although many tools are described as “low cost,” the resource implications vary: anthropometric-only tools may be considered minimal- or near-zero-cost approaches, whereas models requiring laboratory measurements impose greater financial and organizational demands. Therefore, selection of a screening instrument should be aligned with available healthcare infrastructure and screening objectives.

Findings from the included studies suggest that the selection of variables largely depends on the characteristics of the target population. In countries with pronounced economic inequality, such as China, India, and Indonesia, simple questionnaires that do not require biochemical testing are commonly used to minimize costs and facilitate large-scale implementation [23,24,26,28,39,40,44,48,49,52,58,59,62,65]. In settings marked by socioeconomic disparities, variables such as educational level and socioeconomic status are more frequently incorporated [44,54,55,59]. Overall, each tool appears to be designed to achieve acceptable predictive performance within its intended population.

Among the 58 screening tools identified, only 10 included ethnicity as a variable. This was particularly evident in countries characterized by high levels of multiculturalism, such as the United States, Canada, and the United Kingdom, where substantial proportions of the population consist of immigrants [25,31,35,36,41,43,45,63,64]. In the context of increasing global migration, consideration of cultural and demographic diversity may warrant further attention in future tool development.

Population-specific considerations merit further attention. Gestational diabetes history was incorporated in a small number of tools, although women with prior gestational diabetes represent a well-recognized high-risk group for future T2DM. This observation suggests that sex-specific or reproductive history–informed screening approaches remain relatively underrepresented within the mapped literature. Age-related variability also warrants consideration. Although age was included in all identified tools, relatively few studies explicitly examined whether discriminatory ability differed across age strata. Given that T2DM risk increases substantially with advancing age, calibration and threshold optimization for specific age groups may enhance screening precision. Finally, despite representation across 26 countries, certain populations remain underrepresented, including indigenous groups, low-income regions, and specific ethnic minorities. Targeted development and validation of screening tools for these populations may improve equity and contextual applicability.

Although most studies focused on non-invasive screening approaches, a subset of tools incorporated biochemical parameters, including fasting glucose, high-density lipoprotein (HDL), low-density lipoprotein (LDL), triglycerides (TG), and insulin. In certain cases, the addition of fasting glucose and lipid measurements improved the identification of individuals at very high risk of T2DM but did not substantially enhance detection among those at moderate or lower risk [36]. Moreover, in one study, insulin measurement demonstrated lower predictive value compared with glucose-based indicators [63], while its higher cost and technical complexity may limit its practical applicability in routine screening settings. Overall, these observations align with WHO recommendations emphasizing screening approaches that balance predictive performance with affordability and feasibility, particularly in resource-limited healthcare settings.

Where discrimination metrics were reported, performance varied across tools. Several instruments demonstrated AUC values exceeding 0.80, indicating strong discriminative ability, whereas others fell within moderate ranges (0.70–0.75), and a smaller proportion reported lower discrimination. Differences in study design, outcome definitions, and cut-off selection limit direct comparability across tools. In particular, heterogeneity in outcome targets warrants attention: while some instruments were designed to detect undiagnosed T2DM, others aimed to identify prediabetes or broader dysglycemia endpoints. These distinctions are clinically meaningful, as screening for prediabetes primarily supports primary prevention and lifestyle intervention strategies, whereas identification of undiagnosed T2DM may prompt confirmatory diagnostic testing and immediate clinical management.

Importantly, even tools with moderate AUC values may retain substantial utility in population-based screening programs, particularly when high negative predictive value is prioritized. The consistently high negative predictive values reported across several tools suggest suitability for initial population-level screening, where the primary objective is to exclude individuals at low risk and identify those requiring further assessment. Conversely, lower positive predictive values indicate that confirmatory diagnostic testing remains necessary before clinical decision-making, reinforcing the role of these instruments as preliminary risk stratification tools rather than diagnostic tests. Variation in cut-off selection further influences clinical and public health application: lower thresholds typically increase sensitivity at the expense of specificity, potentially leading to higher referral rates for confirmatory testing, whereas higher thresholds prioritize specificity but may miss individuals at moderate risk. The absence of standardized threshold recommendations across populations may therefore complicate direct implementation without local validation.

The use of electronic databases and digital platforms for automated risk identification is increasingly explored as a complementary strategy. Public availability of screening tools through websites or mobile applications may facilitate broader access and earlier engagement. For example, the German screening tool [27] enables individuals to assess their risk electronically. Digital applications allow users to enter personal data, which may be integrated with electronic health records, thereby supporting large-scale preventive initiatives at relatively low cost, particularly when combined with artificial intelligence (AI) approaches [52]. However, this strategy presents challenges, including the need to adapt or redesign tools for digital environments. Tools originally developed for traditional use may not retain equivalent performance when transferred to electronic platforms [75]. Beyond transferability, digital implementation introduces additional practical considerations. Effective deployment of web-based or mobile screening platforms requires user-friendly interface design, clear risk communication, and adaptation to varying levels of health literacy. Integration with electronic health record systems may enhance scalability and automated risk flagging; however, such integration necessitates data interoperability standards and robust data protection frameworks. Issues related to privacy, informed consent, and secure data storage are particularly relevant when personal health information is processed digitally. Moreover, disparities in access to digital technologies—the so-called “digital divide”—may limit equitable implementation, particularly in low-resource or older populations. These factors should be considered when translating screening tools from traditional to digital environments.

In addition to traditional regression-based risk scores, a subset of the identified tools employed machine-learning (ML) approaches for T2DM risk prediction [52,63]. ML-based models can capture complex nonlinear relationships between predictors and may improve predictive performance compared with conventional statistical methods. However, increased algorithmic complexity may reduce interpretability, particularly in black-box models where the contribution of individual predictors is less transparent [76]. In clinical practice, model transparency and explainability remain important for physician acceptance, patient communication, and responsible implementation. Among the studies included in this review, several ML-based models employed different algorithmic architectures, including artificial neural networks, support vector machines, and decision tree algorithms [46,52,62,63]. These models typically relied on structured training and validation procedures, such as train–test splitting or external validation datasets, to evaluate predictive performance. Nevertheless, compared with traditional questionnaire-based screening tools, ML models often require more complex data inputs and computational resources, which may limit their applicability in large-scale population screening. A summary of the methodological characteristics of the machine-learning–based models identified in this review is presented in Supplementary Table S3.

Beyond the initial development of screening models, their application to populations different from those in which they were originally derived requires independent validation, including reassessment of sensitivity and specificity [23,48,54]. These studies represent secondary external validation analyses of previously developed screening tools. As the present review focused on studies proposing or developing screening instruments, such validation studies were not included in the primary synthesis presented in Table 2. Nevertheless, external validation studies provide valuable insight into the cross-population applicability of screening tools. For example, evaluation of the FINDRISC tool in a Greek cohort of 7900 adults aged 35–75 years demonstrated a sensitivity of 81% and specificity of 60% [77], supporting its contextual usability outside its original setting. Similarly, the American Diabetes Association (ADA) Diabetes Risk Test has been validated in Middle Eastern populations, yielding a sensitivity of 78.9% and specificity of 82%, although performance characteristics differed from those observed in the original derivation cohort [78]. Further evidence from an Iranian population showed that the Australian Diabetes Risk Assessment Tool (AUSDRISK) achieved an AUC of 0.67, while FINDRISC demonstrated an AUC of 0.62 for identifying undiagnosed T2DM, indicating moderate discrimination and the potential need for recalibration [79]. Collectively, these findings underscore that screening tool performance may vary across demographic, cultural, and healthcare contexts, highlighting the importance of local validation prior to widespread implementation. In addition, the selection and validation of risk score thresholds represent an important component of practical implementation. Although most studies reported cut-off values used to classify individuals as high risk, the methodological justification for these thresholds was not consistently described. Ideally, threshold determination should be based on ROC curve analysis and evaluation of sensitivity–specificity trade-offs, followed by validation in independent populations and, where appropriate, recalibration to reflect local epidemiological characteristics. Establishing such transparent threshold validation procedures may facilitate the reliable application of screening tools in clinical practice and population-based prevention strategies.

Across the broader literature, anthropometric variables have been extensively investigated as practical and cost-effective indicators of metabolic and functional health. Enhanced anthropometric indices have been reported to improve predictive accuracy for metabolic syndrome beyond traditional measures [80,81], while age-adjusted waist circumference thresholds have been proposed as low-cost approaches for dysglycemia screening [82]. Anthropometric assessment has also been applied in geriatric and metabolic research to estimate muscle mass and support early detection of sarcopenia using both simplified equations and data-driven models [83,84]. More recently, incorporation of anthropometric data into machine learning models has been explored to improve risk prediction for prediabetes [85]. This broader body of evidence provides contextual support for the frequent inclusion of anthropometric variables in the screening tools mapped in the present review.

The public health relevance of early risk identification is further illustrated by epidemiological data from national contexts. In Greece, for example, surveys have reported a considerable prevalence of T2DM among adults, with a substantial proportion of cases remaining undiagnosed [86,87]. Although such epidemiological findings were not systematically analyzed within this scoping review, they provide contextual background that underscores the importance of effective screening approaches.

While previous research has evaluated individual T2DM risk models or specific screening tools, recent systematic reviews have primarily focused on predictive performance, validation procedures, or comparative accuracy within selected models [88,89,90]. In contrast, the present scoping review offers a structured mapping of the breadth and diversity of screening tools developed over the past decades. By identifying 58 distinct tools and documenting their variables, target populations, and implementation characteristics, this review highlights the methodological heterogeneity of the field. Rather than assessing comparative effectiveness, this mapping approach clarifies patterns of variable selection, structural design, and population targeting, thereby contributing to a more comprehensive understanding of the current landscape of T2DM risk assessment tools and their contextual applicability across populations.

Finally, several limitations should be acknowledged. In accordance with scoping review methodology, the objective of the present study was to map the breadth and characteristics of available screening tools rather than to conduct formal methodological quality appraisal or quantitative synthesis of outcomes. Consequently, no structured risk-of-bias assessment was undertaken. Considerable variation was observed in the reporting of validation procedures and performance metrics across studies: while some tools provided discrimination measures such as AUC values and sensitivity/specificity estimates, including external validation in independent populations, others reported limited or incomplete performance data. This heterogeneity restricts direct comparison and precludes definitive conclusions regarding comparative robustness or clinical superiority. Furthermore, the exclusion of secondary validation studies in new populations limits conclusions regarding cross-population generalizability. Cross-population validation is particularly important given ethnic, cultural, and healthcare system differences that may influence tool performance. Future systematic reviews focusing specifically on external validation data would provide valuable guidance for adaptation and contextual implementation. Nevertheless, the review provides a comprehensive mapping of 58 T2DM risk assessment tools across diverse geographical and population contexts. Furthermore, the mapped literature also revealed important gaps, including limited reporting on implementation settings, resource requirements, and real-world applicability. These gaps highlight priorities for future research rather than conclusions regarding tool performance.

## 5. Conclusions

This scoping review provides a comprehensive mapping of screening tools developed for the early identification of adults at high risk of type 2 diabetes. The findings reveal substantial heterogeneity in tool design, variable selection, and implementation context, reflecting adaptation to diverse demographic and healthcare settings. While most tools emphasize feasibility and low-cost application, differences in structure and reporting highlight the absence of standardized approaches across populations. Although this review did not evaluate comparative performance or diagnostic accuracy, it clarifies the current landscape of T2DM risk assessment and identifies important gaps related to validation, reporting consistency, and real-world implementation. Addressing these gaps through cross-population validation studies, harmonized reporting standards, and evaluation of digital integration strategies may strengthen the role of screening tools in primary prevention and public health planning. Policymakers and healthcare planners may therefore consider aligning screening tool selection with local resource availability and population characteristics to optimize implementation and population-level impact.

## Figures and Tables

**Table 1:** PCC framework used to structure the search strategy [16].

PCC Element	Description	Search Terms (Examples)
Population	Adults at risk of developing type 2 diabetes	adult*, population*, individual*, participant*, patient*, undiagnosed
Concept	Screening tools, risk scores, questionnaires, and predictive models used to identify individuals at high risk of developing type 2 diabetes.	screening tool*, risk score*, risk assessment*, questionnaire*, prediction model*, diabetes risk
Context	Early identification of individuals at high risk of type 2 diabetes in general or clinical populations	type 2 diabetes, T2DM, prediabetes, dysglycemia, early diagnosis, risk identification

**Table 2:** Screening variables reported in the 58 eligible studies. The category “Others” includes heterogeneous predictors reported infrequently across studies, such as comorbidities, medication history (e.g., antihypertensive treatment), and specific biochemical or metabolic indicators not consistently categorized across tools. Outcome refers to the primary endpoint targeted for the development of each screening tool.

	Study	Country	Screening Variables																			
			Sex	Age	Height	Weight	Obesity	BMI	Physical Activity	Family History of Diadetes	Hypertension/Blood Pressure	Waist Circumference	Waist-to-Hip Ratio	Ethnicity/Race	Dietary Habits	Smoking Status	Alcohol Consumption	Educational Level	Socioeconomic Status	Gestational Diabetes	Outcome	Others
1	Herman et al., 1995 [17]	USA		✓	✓	✓		✓	✓	✓											Type 2 Diabetes	Macrosomic infant
2	Ruige et al., 1997 [18]	The Netherlands	✓	✓			✓			✓	✓										Type 2 Diabetes	Pain during walking with need to slow down, shortness of breath when walking with people of the same age, frequent thirst, and reluctance to use a bicycle for transportation
3	Baan et al., 1999 [19]	The Netherlands	✓	✓			✓		✓	✓	✓										Type 2 Diabetes	
4	Griffin et al., 2000 [20]	UK	✓	✓				✓		✓	✓					✓					Type 2 Diabetes	Steroid medication
5	Lindstrom and Tuomilehto 2003 [21]	Finland		✓				✓	✓		✓	✓			✓						Type 2 Diabetes	History of high blood glucose
6	Glumer et al., 2004 [22]	Denmark	✓	✓				✓	✓	✓	✓										Type 2 Diabetes	
7	Ramachandran et al., 2005 [23]	India		✓				✓	✓	✓		✓									Type 2 Diabetes	
8	Mohan et al., 2005 [24]	India		✓					✓	✓		✓									Type 2 Diabetes	
9	Schmidt et al., 2005 [25]	USA		✓	✓					✓	✓	✓		✓							Type 2 Diabetes	
10	Aekplakorn et al., 2006 [26]	Thailand		✓				✓		✓	✓	✓									Type 2 Diabetes	
11	Schulze et al., 2007 [27]	Germany		✓	✓				✓		✓	✓			✓	✓	✓				Type 2 Diabetes	
12	Al-Lawati and Tuomilehto 2007 [28]	Oman		✓				✓		✓	✓	✓									Type 2 Diabetes	
13	Wilson et al., 2007 [29]	USA	✓	✓				✓		✓											Type 2 Diabetes	Metabolic syndrome traits (simple clinical model), 2 h post–oral glucose tolerance test
14	Cabrera de León et al., 2008 [30]	Canary Islands		✓						✓	✓									✓	Type 2 Diabetes	Waist-to-height ratio
15	Heikes et al., 2008 [31]	USA	✓	✓	✓	✓		✓	✓	✓	✓	✓		✓						✓	Type 2 Diabetes and Prediabetes	Taking cholesterol medication, high cholesterol
16	Rahman et al., 2008 [32]	UK	✓	✓				✓		✓	✓					✓					Type 2 Diabetes	Prescription of steroids
17	Balkau et al., 2008 [33]	France	✓	✓				✓		✓	✓										Type 2 Diabetes	Fasting glucose, HDL-C
18	Chien et al., 2009 [34]	Taiwan		✓				✓													Type 2 Diabetes	Elevated fasting glucose, triacylglycerol, white blood cell count and a higher HDL-C
19	Hippisley-Cox et al., 2009 [35]	UK	✓	✓				✓		✓	✓			✓							Type 2 Diabetes	Townsend deprivation score, cardiovascular disease, and current use of corticosteroids.
20	Kahn et al., 2009 [36]	USA		✓		✓				✓	✓	✓		✓		✓	✓				Type 2 Diabetes	Glucose, triglycerides, low HDL-C concentration, short stature, high uric acid, rapid pulse
21	Bang et al., 2009 [37]	USA	✓	✓			✓		✓	✓	✓										Type 2 Diabetes	
22	Sun et al., 2009 [38]	Taiwan	✓	✓				✓		✓	✓	✓				✓		✓			Type 2 Diabetes	FPG
23	Gao et al., 2010 [39]	China		✓						✓		✓									Type 2 Diabetes	
24	Xin et al., 2010 [40]	China		✓				✓		✓	✓		✓								Type 2 Diabetes and Prediabetes	
25	Gray et al., 2010 [41]	UK		✓				✓		✓	✓	✓		✓							Type 2 Diabetes and Impaired Glucose Regulation	
26	Chen et al., 2010 [42]	Australia	✓	✓					✓	✓	✓	✓		✓		✓					Type 2 Diabetes	History of high blood glucose level
27	Robinson et al., 2011 [43]	Canada	✓	✓	✓	✓		✓	✓	✓	✓	✓		✓	✓			✓			Type 2 Diabetes	History of high blood glucose level
28	Soewondo and Pramono 2011 [44]	Indonesia	✓	✓			✓				✓	✓				✓		✓	✓		Prediabetes	
29	Gray et al., 2011 [45]	UK	✓	✓				✓		✓	✓			✓							Type 2 Diabetes and Impaired Glucose Regulation	
30	Shankaracharya et al., 2012 [46]	India	✓	✓				✓	✓	✓	✓	✓			✓	✓	✓		✓		Type 2 Diabetes and Prediabetes	High cholesterol
31	Heianza et al., 2012 [47]	Japan	✓	✓				✓		✓						✓					Type 2 Diabetes	FPG, HbA1c
32	Lee et al., 2012 [48]	South Korea		✓						✓	✓	✓				✓	✓				Type 2 Diabetes	
33	Zhou et al., 2013 [49]	China	✓	✓				✓		✓	✓	✓									Type 2 Diabetes	
34	Heianza et al., 2013 [50]	Japan	✓	✓				✓		✓	✓					✓					Type 2 Diabetes	
35	Gray et al., 2013 [51]	Portugal	✓	✓				✓			✓										Type 2 Diabetes and Impaired Fasting Glucose	
36	Choi et al., 2014 [52]	South Korea	✓	✓				✓		✓	✓	✓					✓				Prediabetes	
37	Dugee et al., 2015 [53]	Mongolia	✓	✓	✓	✓		✓	✓		✓	✓			✓	✓					Type 2 Diabetes	History of elevated glucose level
38	Yan et al., 2016 [54]	USA	✓	✓				✓	✓		✓	✓	✓			✓	✓	✓	✓		Type 2 Diabetes	Indian Health Service clinic checkup, past and current health history, cardiometabolic risk, TC, TG, HDL-C, LDL-C, FPG level
39	Zhang et al., 2016 [55]	China	✓	✓				✓	✓	✓		✓			✓	✓	✓	✓	✓		Type 2 Diabetes	PP, HR, TC, TG, HDL-C, LDL-C, FPG
40	Barengo et al., 2017 [56]	Colombia	✓	✓				✓	✓	✓	✓				✓						Type 2 Diabetes and Impaired Glucose Regulation	Fasting glucose, 2 h glucose, history of hyperglycemia, impaired glucose regulation
41	Chen et al., 2017 [57]	China		✓		✓	✓			✓	✓				✓						Type 2 Diabetes	Impaired fasting glucose
42	Zhou et al., 2017 [58]	China	✓	✓				✓	✓	✓	✓	✓									Type 2 Diabetes	History of dyslipidemia
43	Wen et al., 2017 [59]	China		✓				✓	✓	✓		✓	✓			✓		✓			Type 2 Diabetes	FPG
44	Sulaiman et al., 2018 [60]	United Arab Emirates		✓				✓		✓	✓		✓								Type 2 Diabetes	
45	Félix-Martínez and Godínez-Fernández 2018 [61]	Mexico		✓	✓					✓	✓	✓									Type 2 Diabetes	
46	Pei et al., 2019 [62]	China	✓	✓				✓	✓	✓	✓				✓						Type 2 Diabetes	History of cardiovascular disease or stroke, work-related stress
47	Abbas et al., 2019 [63]	USA		✓				✓						✓							Type 2 Diabetes	Plasma glucose and insulin concentrations before glucose intake and at three time-points thereafter (30, 60 and 120 min)
48	Srugo et al., 2019 [64]	USA		✓				✓	✓	✓		✓		✓				✓			Dysglycemia	Delivered infant birthweight
49	Wu et al., 2019 [65]	China		✓			✓			✓	✓	✓									Type 2 Diabetes	
50	Bahijri et al., 2020 [66]	Saudi Arabia	✓	✓						✓		✓									Type 2 Diabetes	History of hyperglycemia
51	Lowe et al., 2020 [67]	Guyana	✓	✓				✓		✓	✓			✓							Type 2 Diabetes	Known diabetes, current treatment
52	Buccheri et al., 2021 [68]	USA		✓								✓									Type 2 Diabetes and Prediabetes	
53	Abbas et al., 2021 [69]	Qatar	✓	✓				✓			✓	✓									Prediabetes	
54	Cho et al., 2021 [70]	South Korea	✓	✓						✓	✓		✓			✓					Type 2 Diabetes	Health check-ups
55	Henjum et al., 2022 [71]	Algeria		✓				✓				✓									Type 2 Diabetes and Prediabetes	
56	Sadek et al., 2022 [72]	Qatar	✓	✓							✓		✓					✓			Type 2 Diabetes and impaired glucose metabolism	History of hyperlipidemia
57	Dong et al., 2022 [73]	China		✓				✓	✓		✓	✓	✓			✓					Type 2 Diabetes and Prediabetes	Sleep duration
58	Alkattan et al., 2024 [74]	Saudi Arabia	✓	✓			✓				✓									✓	Type 2 Diabetes	Dyslipidemia, cardiovascular disorders, hyperplasia of the prostate, ophthalmic disorders, connective tissue disorders, thyroid orders, gastrointestinal disorders, CKD, spondylopathies, refractive errors, nutritional deficiencies, genitourinary infections, and polyneuropathies

HDL-C: high-density lipoprotein cholesterol; FPG: fasting plasma glucose; HbA1c: gylcated hemoglobin; LDL-C: low-density lipoprotein cholesterol; HR: heart rate; TC: total cholesterol; TG: triglyceride; PP: pulse pressure; CKD: chronic kidney disease. ✓ indicates that the variable is included in the screening tool. Narrative text is used in the “Others” column to describe screening variables that are specific to individual models and not commonly included across screening tools.

**Table 3:** Accuracy of the screening tools identified in the 58 eligible studies.

	Study	Country	Accuracy of Screening Tools					
			AUC	Sensitivity (%)	Specificity (%)	(%) Positive Predictive Value	(%) Negative Predictive Value	Comments
1	Herman et al., 1995 [17]	USA		79	65	10		
2	Ruige et al., 1997 [18]	The Netherlands		72	56	6.5	98	
3	Baan et al., 1999 [19]	The Netherlands	0.74 (0.70–0.78) and 0.68 (0.64–0.72)					Two predictive models compared
4	Griffin et al., 2000 [20]	UK	80	77	72			
5	Lindstrom and Tuomilehto 2003 [21]	Finland		78 and 81	77 and 76	13 and 5		Two cohort analyses
6	Glumer et al., 2004 [22]	Denmark	The AROC curve value was 0.804 (95% CI, 0.765–0.838) for the first half of the Inter99 population, 0.761 (95% CI, 0.720–0.803) for the second half of the Inter99 population, and 0.803 (95% CI, 0.721–0.876) for the ADDITION pilot study.	76	72			False-negative risk comparison
7	Ramachandran et al., 2005 [23]	India		76.6, 72.4 and 73.7	59.9, 59.0 and 61.0	9.4, 8.3 and 12.2	97.9, 97.6 and 96.9	Cohort-specific risk threshold
8	Mohan et al., 2005 [24]	India	0.698 (95% CI, 0.663–0.733)	72.5	60.1	17.0	95.1	Overall model accuracy reported
9	Schmidt et al., 2005 [25]	USA	0.75 and 0.78, respectively	40–87	50–86			Metabolic syndrome–based score evaluation
10	Aekplakorn et al., 2006 [26]	Thailand	0.81	76	74			
11	Schulze et al., 2007 [27]	Germany	0.84 in the EPIC-Potsdam and 0.82 in the EPIC-Heidelberg studies.	0.56 in the TUF and 0.45 in the MeSyBePo studies				Cohort-based risk gradient analysis
12	Al-Lawati and Tuomilehto 2007 [28]	Oman		78.6 and 62.8	73.4 and 78.2			Cohort-specific cut-off threshold
13	Wilson et al., 2007 [29]	USA	0.85					
14	Cabrera de León et al., 2008 [30]	Canary Islands						Age-stratified screening performance
15	Heikes et al., 2008 [31]	USA	0.85	88 and 75	75 and 65	14 and 49	99.3 and 85	Diabetes and prediabetes detection models
16	Rahman et al., 2008 [32]	UK	0.745					
17	Balkau et al., 2008 [33]	France	0.713 for men and 0.827 for women					
18	Chien et al., 2009 [34]	Taiwan		52	78			
19	Hippisley-Cox et al., 2009 [35]	UK						Sex-specific risk model performance
20	Kahn et al., 2009 [36]	USA	0.71 (95% CI, 0.69–0.73) and 0.79 (95% CI, 0.77–0.81)	69 and 74	64 and 71			Basic vs. enhanced scoring systems
21	Bang et al., 2009 [37]	USA		79	67	10		Positive likelihood ratio reported
22	Sun et al., 2009 [38]	Taiwan	0.848 (95% CI, 0.829–0.868)	72.26% and 76.64%	82.84% and 76.00%	19.87 and 19.47		Separate cohort validation
23	Gao et al., 2010 [39]	China		85.6 and 75.5	21.1 and 43.6			Sex-specific model evaluation
24	Xin et al., 2010 [40]	China		74.6 and 65.3	71.6 and 72.5	23.6 and 33.2	96.0 and 90.7	Diabetes and combined outcome models
25	Gray et al., 2010 [41]	UK		72	81			
26	Chen et al., 2010 [42]	Australia	0.78 (95% CI, 0.76–0.81)	74.0	67.7	12.7		Independent cohort validation
27	Robinson et al., 2011 [43]	Canada	0.806	72.7	68.1			
28	Soewondo and Pramono 2011 [44]	Indonesia						
29	Gray et al., 2012 [45]	UK	70.1 (95% CI, 68.4–71.7)					
30	Shankaracharya et al., 2012 [46]	India		99.5	99.07			Classification accuracy reported
31	Heianza et al., 2012 [47]	Japan	0.771 (95% CI, 0.758–0.784)	72.7	68.1	6.4	98.8	
32	Lee et al., 2012 [48]	South Korea	0.73	81	54	6		Validation dataset performance metrics
33	Zhou et al., 2013 [49]	China	0.748 (95% CI, 0.739–0.756) in the exploratory population, 0.725 (95% CI, 0.683–0.767) in validation 1, and 0.702 (95% CI, 0.680–0.724) in validation 2	92.3 and 86.8	35.5 and 38.8			Dual validation cohorts
34	Heianza et al., 2013 [50]	Japan	0.887 (95% CI, 0.871–0.903)	83.7	79.0			
35	Gray et al., 2013 [51]	Portugal	0.74 (95% CI, 0.72–0.77)	73.2 and 69.1	55.5 and 63.3	27.0 and 38.0	90.2 and 86.2	Cross-sectional and prospective validation
36	Choi et al., 2014 [52]	South Korea		74 and 70	70 and 61			
37	Dugee et al., 2015 [53]	Mongolia	0.77 (95% CI, 0.71–0.82)	81.4	58.9	10.9	98.1	Defined cut-off threshold
38	Yan et al., 2016 [54]	USA	0.68					
39	Zhang et al., 2016 [55]	China	0.768 (95% CI, 0.760–0.776)	66.7	74.0			
40	Barengo et al., 2017 [56]	Colombia	0.74 (95%CI, 0.70–0.79)	73	67	10.6	97.9	
41	Chen et al., 2017 [57]	China	0.705 and 0.754	70.5 and 63.1	60.4 and 75.9	2.5 and 2.5	99.3 (noninvasive model)	Noninvasive vs. laboratory model
42	Zhou et al., 2017 [58]	China	0.723 (95% CI, 0.710–0.735)	67.9	67.8			
43	Wen et al., 2017 [59]	China	0.686	74.32	58.82			
44	Sulaiman et al., 2018 [60]	United Arab Emirates	0.82 (95% CI, 0.78–0.86).	75.4	70	45.3	89.6	
45	Félix-Martínez and Godínez-Fernández 2018 [61]	Mexico	0.70 and 0.66	0.74 and 0.76	0.62 and 0.55			National survey–derived models
46	Pei et al., 2019 [62]	China	94.2%, 94.0%, 94.2%, and 94.8%.					
47	Abbas et al., 2019 [63]	USA		80.09				Average model accuracy reported
48	Srugo et al., 2019 [64]	USA	0.69 and 0.92					Sex-specific prediction models
49	Wu et al., 2019 [65]	China	0.654 (95% CI, 0.629–0.680) and 0.684 (95% CI, 0.662–0.705)					Sex-stratified model analysis
50	Bahijri et al., 2020 [66]	Saudi Arabia	0.76 (95% CI, 0.73–0.79)	0.7	0.7			Alternative cut-off thresholds evaluated
51	Lowe et al., 2020 [67]	Guyana	0.812	88.2	43.7	38.1	90.4	Accuracy reported for defined threshold
52	Buccheri et al., 2021 [68]	USA		65	73			
53	Abbas et al., 2021 [69]	Qatar	80% (95% CI, 0.78–0.83)	86.2	57.9	49.5	89.75	
54	Cho et al., 2021 [70]	South Korea	0.760 (95% CI, 0.752–0.767)	82.7	58.2			
55	Henjum et al., 2022 [71]	Algeria	0.82	89	65	28	97	
56	Sadek et al., 2022 [72]	Qatar	0.870 (95% CI, 0.843–0.896)	84.6	76.2	28.7	97.7	
57	Dong et al., 2022 [73]	China	0.812 and 0.822					Logistic regression vs. ML model comparison
58	Alkattan et al., 2024 [74]	Saudi Arabia	0.803 (95% CI, 0.779–0.826)					

AUC: area under the curve; CI: confidence interval; EPIC: European Prospective Investigation into Cancer and Nutrition; TUF: Tübingen Family Study; MeSyBePo: Metabolic Syndrome Berlin Potsdam; AROC: area under the receiver operating characteristic; ML: machine learning; Blank cells indicate information not reported in the original source study and should not be interpreted as zero or not applicable.

## Data Availability

All data analyzed during this study are included in this manuscript.

## Supplementary Materials

The following supporting information can be downloaded at: https://www.mdpi.com/article/10.3390/healthcare14070839/s1, Table S1. PRISMA-ScR-Fillable-Checklist [15]; Table S2. Complete search strategies used in each database; Table S3. Machine-learning–based models [46,52,62,63].

## Abbreviations

The following abbreviations are used in this manuscript:

DM	Diabetes mellitus
T2DM	Type 2 diabetes mellitus
PDM	Prediabetes
AUC	Area Under the Curve
AROC/ROC	(Area under the) Receiver Operating Characteristic
CI	Confidence Interval
PPV	Positive Predictive Value
NPV	Negative Predictive Value
LR	Logistic Regression
ML	Machine Learning
BMI	Body Mass Index
WC	Waist Circumference
WHR	Waist-to-Hip Ratio
BP	Blood Pressure
HR	Heart Rate
PP	Pulse Pressure
FPG	Fasting Plasma Glucose
HbA1c	Glycated Hemoglobin
HDL	High-Density Lipoprotein
LDL	Low-Density Lipoprotein
TG	Triglycerides
TC	Total Cholesterol
PRISMA	Preferred Reporting Items for Systematic Reviews and Meta-Analyses
PRISMA-ScR	PRISMA Extension for Scoping Reviews
PICO	Population, Intervention, Comparison, Outcome
WHO	World Health Organization
IDF	International Diabetes Federation
EPIC	European Prospective Investigation into Cancer and Nutrition
TUF	Tübingen Family Study
MeSyBePo	Metabolic Syndrome Berlin Potsdam
CKD	Chronic kidney disease
