# Supplementary material for: Screening Tools for Early Identification of Adults at High Risk of Type 2 Diabetes: A Scoping Review
Source: Healthcare (Basel). 2026 Mar 25;14(7):839. doi: 10.3390/healthcare14070839 (PMC13072748; doi:10.3390/healthcare14070839)
Supplement: Supplementary file 1 [file healthcare-14-00839-s001.zip › Table S2. Complete search strategies used in each database.pdf]

Table S2. Complete search strategies used in each database*.

Database	Search Date	Full Search Strategy
**Scopus**	7 January 2026	TITLE-ABS-KEY(adult*) AND TITLE-ABS-KEY("risk score*" OR "risk assessment" OR "screening tool*" OR "screening questionnaire*" OR "prediction model*" OR "risk calculator*" OR "diagnostic tool*") AND TITLE-ABS-KEY("type 2 diabetes" OR T2D OR NIDDM)
**ScienceDirect**	7 January 2026	adult* AND ("risk score*" OR "screening tool*" OR "prediction model*" OR "risk calculator*") AND ("type 2 diabetes" OR T2D)
**Web of Science Core Collection**	7 January 2026	(adult* AND ("risk score*" OR "screening tool*" OR "prediction model*" OR "risk calculator*") AND ("type 2 diabetes" OR T2D OR NIDDM)) AND TS=("early diagnos*" OR undiagnos* OR prediabet* OR "high risk")
**PubMed (MEDLINE)**	6 January 2026	((adult*[Title/Abstract] OR individual*[Title/Abstract] OR subject*[Title/Abstract]) AND (tool*[Title/Abstract] OR "risk score"[Title/Abstract] OR screen*[Title/Abstract] OR scor*[Title/Abstract] OR predict*[Title/Abstract] OR questionnaire*[Title/Abstract]) AND ("Type 2 Diabetes Mellitus"[MeSH] OR "type 2 diabetes"[Title/Abstract] OR T2DM[Title/Abstract] OR NIDDM[Title/Abstract] OR prediabet*[Title/Abstract] OR "high risk"[Title/Abstract] OR identif*[Title/Abstract] OR detect*[Title/Abstract] OR validat*[Title/Abstract]))

*Search strategies were adapted to the syntax and search functionality of each database while maintaining the same conceptual structure based on the Population–Concept–Context (PCC) framework.
