# Supplementary material for: Screening Tools for Early Identification of Adults at High Risk of Type 2 Diabetes: A Scoping Review
Source: Healthcare (Basel). 2026 Mar 25;14(7):839. doi: 10.3390/healthcare14070839 (PMC13072748; doi:10.3390/healthcare14070839)
Supplement: Supplementary file 1 [file healthcare-14-00839-s001.zip › Table S3. Machine-learning–based models.pdf]

Table S3. Methodological characteristics of machine-learning–based models identified in the included studies*.

Study	ML architecture	Validation approach	Key predictors
**Shankaracharya et al., 2012 [46]**	Mixture of Experts neural network	Train–test split (training dataset n=1104; validation dataset n=311)	clinical and metabolic variables from hospital records
**Choi et al., 2014 [52]**	Artificial Neural Network (ANN); Support Vector Machine (SVM)	Internal validation using training dataset (KNHANES 2010) and external validation dataset (KNHANES 2011)	demographic, anthropometric, and clinical predictors (e.g., age, BMI, blood pressure)
**Pei et al., 2019 [62]**	J48 decision tree algorithm	Model development using filtered cross-sectional dataset	demographic, lifestyle, and clinical variables (e.g., age, BMI, hypertension, family history)
**Abbas et al., 2019 [63]**	Support Vector Machine (SVM) with feature selection (mRMR)	Train–test split with holdout validation dataset	OGTT-derived glucose features and demographic variables

*Only studies that explicitly reported the machine-learning architecture were included in this table. One study describing a machine-learning–based screening model did not specify the algorithm used and was therefore not included.
